# Annotations

**Published:** 1896-07-11

**Authors:** 


### ANNOTATIONS.

### Cholera in Egypt.

THE news from Egypt in regard to cholera seems to us to be more serious than some people imagine. The population of all Egypt is, perhaps, half as large again as that of London, certainly not more, and yet we find that the returns last week give the cases of cholera as 1,937 and the deaths as 1,566. We may be quite certain then that Egypt is suffering from a very severe outbreak of the disease just at the most dangerous time of the year. What, however, makes the position of affairs all the more serious is the attitude of the people in regard to the sanitary measures which are necessary for the control of the epidemic. Reports have continued to arrive as to the difficulties experienced by the sanitary officials in securing the isolation of the cases attacked, and even in obtaining information as to the presence of the disease. Nothing short of the necessity for an order for burial seems to be of much effect in bringing many of the cases to light, and when we find that 80 per cent. of the reported cases die, we cannot but see that many of the milder cases are still escaping all sanitary control. Herein lies the gravity of the position, for while the slighter cases are hidden away in the towns and villages, it is the most difficult thing in the world to impose any effectual check on the progress of the epidemic. In view of all that is known as to the common relation of cholera with wars and great movements of population, and bearing in mind what is taking place in Egypt at the present time, it is impossible to repress feelings of anxiety in regard to the future of this great outbreak of cholera.

### "Free from Infection."

THE question of the length of time during which a patient who has suffered from scarlet fever continues to be infectious is a matter of very great interest to the public; and since the Birmingham Corporation was last year mulcted in £50 as compensation for damages caused, as it was alleged, by the premature discharge of a patient after scarlet fever, the same question has become a matter of the most vital interest to sanitary authorities; for if all of them were made to pay in like fashion for their "return cases" the loss would amount to thousands of pounds per annum. In the good old times, the good old simple plan was to go by the "peeling," and maintain that so long as there was desquamation there was danger, and that if a case which had finished peeling still appeared to carry infection there must have been a mistake somewhere. Probably this is the opinion of nine out of ten doctors even now. There is, however, much reason to believe that this opinion is erroneous. In a discussion recently held on the subject by the Society of Medical Officers of Health, Dr. Boobbyer said he had long held the opinion that desquamation, in the later stages of convalescence at any rate, was in itself of no pathological importance, adding that he could give many examples out of his own experience of desquamating patients who had mixed freely with presumably susceptible persons without communicating the disease, and referring to the well-known instance of the discharge of 120 desquamating patients from the Leicester Hospital without producing a single secondary case. Dr. Millard, of the City Hospital, Birmingham, who had been working at the subject for some time, said that he regarded desquamation as practically of no value at all as an indicator, and did not think it was a source of danger. Again, if we turn to the recently issued report of the Metropolitan Asylums Board, we find that in actual practice many cases are discharged before desquamation is over. This would agree with the opinions expressed at the meeting above referred to, and elsewhere, viz., that it is to inflammatory conditions of mucous membranes, and, in a certain degree, of the skin, to which we must look as the starting point of infection, and not to the mere continuance of desquamation. So much for the scientific aspect of the question. What the public should recognise, and what doctors must admit, is that we do not know, and cannot know with any approach to certainty, when a patient is free from the power of setting up scarlet fever in others, and that hospitals can no more undertake to return patients "free from infection" than they can promise to return them alive.

### Holidays and Heredity.

How to take a holiday? where to take a holiday? and when to take a holiday? are always questions of great interest, but as the July days drift on towards August they become problems of the first magnitude. Perhaps it is not so easy as some would think to define what we mean by holiday. To different people it means a different thing, but it always involves the idea of change. This is but a platitude. But why should a man so crave for change? and why should one who apparently has got all he wants, whose work by practice has become easy, whose wife expects him at a certain hour, whose dinner is always ready, whose servants have at last, after many vicissitudes, settled down into regular ways—why, indeed, should he spend hours over "Bradshaw" in delightful anticipation of upsetting all this well-ordered life and going down by crowded train to frowsy lodgings all for the sake of change? Dr. Louis Robinson some years ago by watching babies and their ways came to the conclusion, not that they had in them a dash of the old Adam, but that the root-motive of their actions went back much further still; in other words, that they had within them a strong strain of the real old original monkey from which they were descended. Why else should a new-born child hold on so vigorously to a walking stick as to allow itself by the grip of its tiny hands to be raised up and swung clear of the bed on which it lay? Clearly the new-born infant, uncivilised as yet by soap and water, clothes, and baby bottles, thought itself again a little monkey, swinging by a branch in its native forest! So fertile a scientific imagination as that of Dr. Robinson clearly could not end, as it had begun, in describing the vagaries of babies. Working on the same lines, he now tells us in the *National Review* why it is that we crave for holidays and change. It is a most respectable craving, does not go back half so far as monkeys, and, in fact, originates from the habits developed during that long period in man's history when he was a nomadic creature always on the wander. In those days man's life was one of constant change; and the more monotonous man's life is now, the more does he tend, when the occasion arises, to spring back with a rebound to the habits of a former state, in which, whatever the hardships of his lot, at least he had change of scene.

